# Aromatherapy for Labour Pain Management: Umbrella Review

**DOI:** 10.3390/healthcare14050573

**Published:** 2026-02-25

**Authors:** Nicole Breuninger, Harald Abele, Joachim Graf

**Affiliations:** 1 Department of Midwifery Science, Institute for Health Science, University Hospital Tuebingen, 72076 Tuebingen, Germany; harald.abele@med.uni-tuebingen.de (H.A.); joachim.graf@med.uni-tuebingen.de (J.G.); 2 Department of Women’s Health, University Hospital Tuebingen, 72076 Tuebingen, Germany

**Keywords:** aromatherapy, labour pain, essential oil, inhalation, analgesia, complementary medicine, alternative medicine, midwifery, intrapartum pain, pain management, lavender oil, rose oil, umbrella review

## Abstract

**Highlights:**

**What are the main findings?**

- Aromatherapy can possibly reduce labour pain. However, the quality assessment revealed an existing risk of bias.
- Questions remain as to which oils, application methods, or dosages are most useful.

**What are the implications of the main findings?**

- Midwives experienced in the practice may use aromatherapy according to their clients’ wishes.
- Research must be conducted to derive more concrete guidelines.

**Abstract:**

**Background/Objectives**: Aromatherapy is widely used in midwifery, with pain relief during labour being a common objective. This umbrella review consolidates evidence from systematic reviews and meta-analyses on its efficacy for this purpose. **Methods**: Following PRISMA guidelines, nine systematic reviews and meta-analyses published between 2011 and 2023 were included, encompassing 127 primary studies. Populations, interventions, comparators, and outcomes were systematically extracted, and methodological quality was assessed using AMSTAR 2. **Results**: Lavender and rose were the most commonly used essential oils. Application methods included inhalation, massage, and bathing. Across reviews, aromatherapy showed statistically significant effects on labour pain reduction, particularly during the early stages of labour. However, high heterogeneity, low methodological quality of many reviews, and inconsistent reporting limit the overall strength of the evidence. No adverse effects were reported. **Conclusions:** The study results suggest that aromatherapy could be an effective, non-invasive intervention for labour pain. However, due to the low methodological quality of the studies and the correspondingly low strength of evidence, these results cannot yet be applied to the general population. This means that no general recommendation for use can be made at this time. Available reviews did not identify an increase in adverse maternal or neonatal outcomes, but safety monitoring and reporting were often limited by low review quality and inconsistent methods. Future high-quality randomized trials and evidence-based clinical guidelines are needed.

## 1. Introduction

Aromatherapy, the therapeutic use of essential oils, has a long history in both traditional medicine and modern obstetric practice. Its origins can be traced back thousands of years, with aromatic plant extracts employed for medicinal, spiritual, and cultural purposes across ancient civilizations [1]. Throughout history, midwives in particular have used aromatic oils to support women during pregnancy, childbirth, and the postpartum period [2]. The modern resurgence of aromatherapy began in early 20th-century Europe, when the term itself was coined by Renée Gattefossé [3]. The practice has since steadily gained ground across healthcare professions in the treatment of anxiety, nausea, depression, and pain [1]. Despite its widespread use, its role within evidence-based practice remains controversial, as scientific studies provide heterogeneous and sometimes contradictory results.

Labour pain is widely recognized as one of the most intense forms of pain experienced by humans. While it originates physiologically from uterine contractions and the stretching of birth canal tissues, its perception is shaped by psychological, cultural, and individual factors [4]. Individualized pain management is therefore a primary concern in maternity care. Established pharmacological interventions such as epidural anaesthesia and opioid analgesia are effective but often rejected by women due to their high invasiveness and fear of side effects. Consequently, there has been growing interest in complementary and non-pharmacological approaches to support women in labour in both hospital and home settings. These approaches—such as massage, relaxation techniques, and aromatherapy—fit within a woman-centred model of care that emphasizes autonomy, empowerment, and minimizing unnecessary interventions. Studies show midwives internationally view complementary and alternative medicine (CAM) and aromatherapy in particular as “philosophically congruent” [5] (p. 1) to their profession, highlighting it as an alternative to conventional interventions that empowers those treated as well as furthering the professional autonomy of midwives [5]. A study by Burns et al. (2000) showed remarkable user satisfaction among women who used aromatherapy during childbirth, with only 14% of users deeming it unhelpful [6]. During an eight-year study conducted in a British teaching hospital, where women in labour were routinely offered aromatherapy, pethidine use reportedly declined from 6% to 0.2% of women (ibid.). While uncontrolled and unblinded, these observations indicate a real-world effect of clinical aromatherapy endorsement on opioid use during labour.

Aromatherapy in labour can be applied in a variety of ways and tailored to individual and situational needs. Its exact mechanism of action remains unknown; however, it is widely accepted that sensory stimulation of the olfactory bulb, which is closely connected to emotional processing centres of the brain, plays a large role, potentially combined with mucosal or transdermal molecular uptake [7]. Oils are selected based on the intended effect and personal preference. Table 1 shows a selection of popular oils used in midwifery and their respective profiles, based on a selection of aromatherapy handbooks and journal articles.

These oils are believed to reduce pain perception and promote relaxation, potentially lowering anxiety and stress, which in turn may positively influence the course of labour. Such effects resonate with the “working with pain” paradigm in midwifery, which frames labour pain as a natural and meaningful process that can be supported rather than suppressed [4] (p. 1). This is in contrast to the “pain relief” paradigm, which views pain primarily as a problem to be eliminated. Aromatherapy, therefore, sits at the intersection of cultural values, clinical practice, and scientific evaluation [4].

The mode of action, which has not yet been sufficiently understood or researched, is thought to be based on a complex neurobiopsychosocial mechanism. Essential oils (such as lavender, rose, or jasmine) act on the brain via olfactory stimuli, influencing hormones, reducing anxiety, and relaxing the muscles. This activates the limbic system: fragrances enter the limbic system, the centre for emotions and memory, directly through the nose. This can trigger rapid relaxation and reduce anxiety. The scent stimulates the brain to release pain-relieving neurotransmitters and hormones such as endorphins and serotonin, which can alleviate labour pains [16,17]. Study results suggest that aroma inhalation can lower cortisol levels, leading to a decrease in stress and anxiety and thus reducing pain perception. Furthermore, essential oils can interact with cell membranes, thereby dampening the pain signals that are transmitted to the brain [16,17,18,19].

Despite anecdotal and clinical support, the evidence for aromatherapy in labour remains fragmented. Systematic reviews and meta-analyses suggest possible benefits, but inconsistencies in study designs, oil types, application methods, and outcome measures limit the reliability of their conclusions. There is also a need to address methodological weaknesses such as inadequate blinding and incomplete reporting, which complicate efforts to establish a robust evidence base.

No scientific consensus has yet been reached concerning the effectiveness of aromatherapeutic intervention for labour pain management, with some authors cautioning against its light-hearted endorsement while indications and contraindications remain disputed [20]. While generally perceived as “natural” and “safe”, essential oils are highly concentrated and can trigger adverse reactions, most commonly contact dermatitis due to skin irritation (due to improper dilution), sensitization (due to prolonged or repeated use of an oil), or allergic reaction [20]. There is little evidence appropriate for conclusive safety assessments specifically for use during pregnancy and birth, and incidences cannot be conclusively established; adverse effects may also be underreported [20]. Aromatherapy experts caution practitioners to take regular breaks from aromatherapeutic interventions during labour to avoid sensory overstimulation and sensitization. Proper dilution with a carrier oil of 2% or lower and patch testing on an easily rinsed body part, like the forearm, are commonly highlighted as measures that can reduce the risk of adverse effects [21]. Due to the lack of evidence, these recommendations should be considered with caution in practice.

The aim of this umbrella review is to synthesize the findings of existing systematic reviews and meta-analyses on aromatherapy for labour pain management. The hypothesis that aromatherapy could have a positive effect on the experience of labour pain will be explicitly considered. By critically evaluating the quality of these reviews and summarizing their results, this paper seeks to provide a comprehensive overview of current knowledge. In doing so, it contributes to the broader discussion of how complementary therapies such as aromatherapy can be responsibly integrated into evidence-based maternity care.

## 2. Materials and Methods

This umbrella review followed PRISMA guidelines and included systematic reviews and meta-analyses of aromatherapy for labour pain. Inclusion criteria were guided by the PICO framework: *Participants* were women in labour; *interventions* included aromatherapy using any essential oil and administration method; *comparators* were control groups of labouring women receiving placebo or standard care, and the primary *outcome* was labour pain intensity; other proposed effects of intrapartum aromatherapy will be discussed as secondary outcomes. As an umbrella review, only systematic reviews with or without meta-analysis were eligible for inclusion. The search was additionally restricted to papers written in either German or English. This umbrella review searched two databases, PubMed and Google Scholar. The search was limited to two databases because it was assumed that journals with a biomedical or health science background that were not included in Medline or PMC were more likely to have quality deficits. All relevant obstetrics journals or journals covering obstetrics topics are listed in PubMed. The authors are aware that reviews published in journals that do not focus on biomedicine may have been overlooked. In accordance with the quality requirements for systematic literature reviews, an internal a priori protocol was drawn up (not registered), from which no relevant deviations occurred.

Following the guidelines for systematic research, the following search string was entered into the PubMed scientific search engine multiple times over the course of May and June 2025: *(aromatherapy) AND ((labour pain) OR (labor pain))*. Filters were therefore applied to select only reviews, systematic reviews, and meta-analyses that were published between 2000 and 2025. To search Google Scholar, the following search string was used: *“aromatherapy for labour pain” OR “aromatherapy for labor pain” in title: review*.

**Figure 1:**
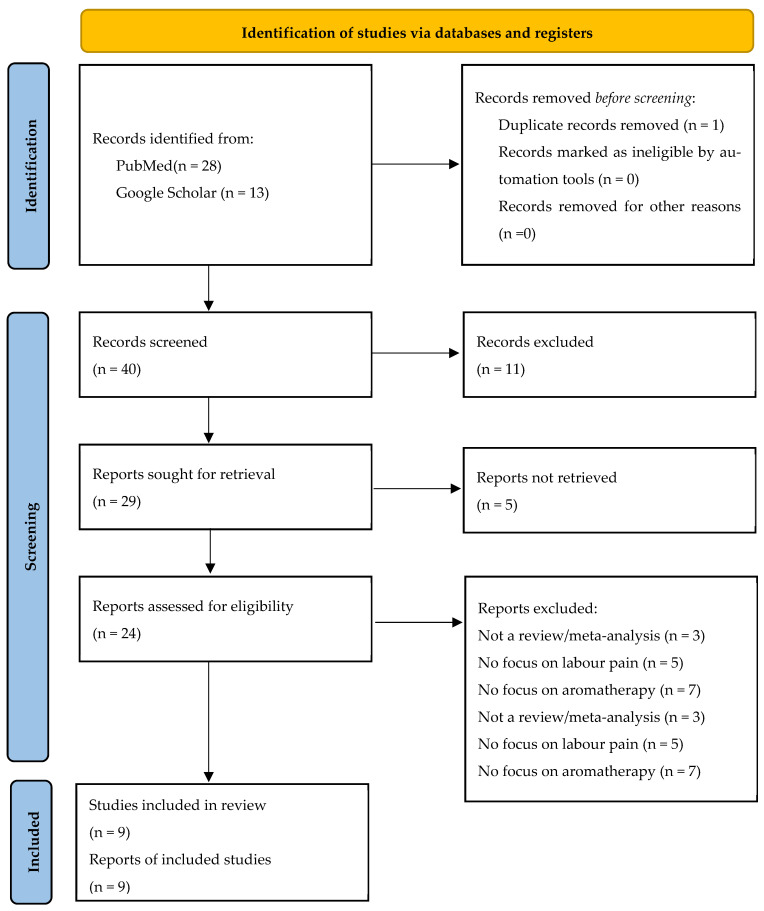
PRISMA scheme used for review selection. Source: [22].

Filters were applied equivalently to those detailed for PubMed. Combined, the two scientific databases returned a total of 41 hits, as seen in the PRISMA scheme (Figure 1). After removing primary trials, comments, monographs, and duplicates, the remaining publications’ abstracts were screened for relevance. Through this, 16 publications were found to be eligible for full-text review, 4 of which had to be excluded because they could not be accessed in English or German. The remaining papers were read in full.

Overall, nine publications were identified as a match for this study’s PICO scheme and consequently entered into the umbrella review. Additional manual searching was conducted by screening the reference lists of included reviews to ensure that no major eligible publication had been missed due to indexing inconsistencies. No additional reviews meeting the inclusion criteria were identified through this process. The inclusion criteria were defined clearly prior to conducting the literature search, and no changes were made post hoc. For best practice, a full list of all excluded publications and their respective exclusion criteria is available in Appendix A.1.

## 3. Results

### 3.1. Characteristics of Included Studies

#### 3.1.1. Population

The included reviews were conducted primarily in the Middle East. Most of the reviews included only randomized controlled trials (RCTs), with some including non-randomized studies of interventions (NRSIs) and one review failing to report on the design of their included trials. Most included reviews restricted their populations to low-risk patients, excluding those with high-risk pregnancies, preterm labour, breech presentation, and multiples. This focus on “typical cases” is common in complementary and alternative medicine (CAM) research, as methods such as aromatherapy are primarily meant to support physiological labour. Only Liao et al. (2021) [23] restricted their selection of primary studies by parity, though two more explicitly described their population as primarily primigravid. Table 2 shows data pertaining to the studies’ origin, population, and sample size of primary studies. The full selection process is depicted in Figure 1. A data extraction sheet was developed to consistently capture key information from each included review, including authorship, publication year, number and design of included studies, type of intervention and comparator, target population, primary and secondary outcome measures, and overall conclusions. One author conducted data extraction as well as an assessment of methodological quality using AMSTAR 2.

#### 3.1.2. Application Methods and Essential Oils

The reviews largely included trials with varying application methods, differing in route of administration, duration of intervention, and dose. However, in most primary studies, aromatherapy was administered through some type of inhalation. The second most popular application method was massage using a carrier oil, into which the essential oil was added as the active ingredient. Detailed descriptions of intervention protocols were scarce. A wide selection of essential oils was represented in the reviews, with at least 14 individual oils mentioned across all primary trials. Across all reviews, lavender essential oil was the most popular, appearing as an intervention in the most primary trials and being discussed in all but one review [31]. It is followed by rose, mixed oils, and jasmine oil. Data on application methods and essential oils represented by reviews and primary trials are depicted in Table 3.

#### 3.1.3. Control Groups

All reviews included primary trials that used either placebo or standard care control groups, each offering different advantages. Placebo controls help distinguish the specific effects of essential oils from those of accompanying procedures such as massage or breathing, although true blinding is difficult due to the recognizable scents involved. Standard care controls, while less effective at isolating confounders, provide more direct clinical relevance by comparing aromatherapy with established practices; however, “standard care” varied widely across settings and was often poorly described. Across the included reviews, reporting on control conditions was inconsistent: several papers misclassified controls, some trials used active comparators, and others failed to specify control procedures altogether. Key details—such as access to additional pain relief and timing of pain assessments in control groups—were frequently missing, limiting interpretability and comparability across studies. Table 4 shows data on control groups and secondary outcomes present in the literature sample.

#### 3.1.4. Outcomes

The primary outcome in all included reviews was labour pain intensity, measured via well-established tools such as the Numerical Pain Rating Scale (NRS) or the Visual Analogue Scale (VAS). The exact timing of these measurements was underreported and inconsistent across the studies. Anxiety was the most frequently examined secondary outcome, assessed in four of the nine reviews, reflecting its close connection to pain perception and overall well-being during labour. Aromatherapy is known to reduce anxiety in other clinical contexts, which likely contributed to research interest in this outcome. Anxiety was measured using validated tools such as the Visual Analogue Scale for Anxiety (VASA) and the State–Trait Anxiety Inventory (STAI). Other secondary outcomes included Apgar scores, labour duration, delivery mode, emergency caesarean section, use of analgesia, and neonatal unit admissions, providing broader insight into maternal and neonatal safety. However, because these outcomes were not required for inclusion and were inconsistently reported across primary studies, the evidence remains limited, and conclusions must be interpreted with caution.

### 3.2. Main Results

Eight of the nine included reviews concluded that aromatherapy is able to lower the intensity of pain experienced during labour and birth. The only exception, the study by Smith et al. (2011) [31], was the oldest of the included papers, and the authors failed to recruit enough primary trials that fit their inclusion criteria to make any inferences from their synthesis. Results showed small to moderate effect sizes with high heterogeneity in most of the meta-analyses. Findings concerning the comparative effectiveness of different essential oils were limited, as most reviews did not perform sub-analyses investigating this question. If authors highlighted the effect of any oil in particular, it was lavender oil, which was also the most well-represented oil in the primary research.

Four of the included reviews investigated if and how the analgesic effect of aromatherapeutic intervention differed across labour stages; different papers showed an effect in the latent, early-active, and late-active (transition) stages of labour, but did not agree on any time points of labour that benefit most or least from aromatherapy. One review reported consistent effects across all labour stages.

Only one review compared the effectiveness of different application methods, reporting indications of a possible advantage of inhalation aromatherapy over massage-based approaches.

All four reviews investigating anxiety levels concluded aromatherapy to be an effective treatment to reduce this common negative factor during labour, showing support for the notion that aromatherapy works at least in part by calming the mind and thus helping women feel more in control of their labour experience.

None of the nine reviews discussed here showed any negative effects of aromatherapy. Neither maternal nor foetal/neonatal safety indicators, such as emergency caesarean incidence, Apgar scores, or admission rates to NICU facilities, were influenced by aromatherapeutic treatment during labour. Eight reviews concluded aromatherapy to be safe as an intervention but stressed the need for more well-designed, larger studies to be concluded; the authors of the Cochrane review did not find enough evidence to make judgements on any of their primary or secondary outcomes, including those concerning safety. A per-review summary of the main findings and relevant statistical figures can be seen in Table 5.

### 3.3. Qualitative Assessment Using AMSTAR2

Umbrella reviews provide valuable high-level insight but are particularly susceptible to accumulating bias from the systematic reviews they include, a risk known as compound bias. To mitigate this, the methodological quality of each review must be rigorously evaluated. AMSTAR 2, designed for reviews of randomized and non-randomized studies, assesses 16 methodological domains and assigns an overall confidence rating (high, moderate, low, or critically low) rather than a numerical score, allowing for a more nuanced appraisal of credibility [32]. Its seven critical domains carry particular weight in determining overall quality, though reviewers are encouraged to judge their relevance case by case. The included reviews were therefore assessed using AMSTAR 2, with individual item ratings and overall confidence levels summarized in Table 6. This appraisal provides the foundation for interpreting the reliability and strength of the evidence synthesized in this umbrella review. Items 3 (justification of study design restrictions), 7 (providing a list of potentially eligible studies and the respective reasons for their ultimate exclusion), 10 (investigation into primary studies’ funding), and 15 (investigation of risk of publication bias) showed the least compliance, leading to concerns surrounding the quality of the studies at hand to be examined in the Discussion.

## 4. Discussion

This umbrella review’s conclusions apply primarily to low-risk women experiencing low-risk labour, as both the included reviews and their underlying trials largely excluded high-risk populations. Future research should explore potential benefits for women with risk factors, who could benefit a lot from non-invasive pain and anxiety treatment, yet remain understudied in both obstetric and complementary medicine research. Geographically, most included trials originated from the Middle East, with five of the nine reviews conducted in Iran or Turkey. This concentration limits generalizability to other populations and may also have influenced the oils and application methods studied. Cultural preferences play a notable role in aromatherapy practices, as seen in the common use of frankincense in Middle Eastern research, which is not discussed in any of the examined German midwifery literature (see Table 1 and Table 3). Other plant families, such as Lamiaceae, Umbelliferae, and Asteraceae, may indeed offer pain-relieving effects but were not represented in the literature at hand, possibly because they are not traditionally used in the regions where the primary studies were conducted.

The selection of oils in the reviewed trials was often not individualized. In practice, aromatherapy typically considers each woman’s physical and emotional state as well as personal preference, yet most studies used standardized oils and protocols. Consequently, it remains unclear whether individualized oil selection could enhance analgesic or secondary effects, such as reducing stress or nausea.

As this umbrella review relied on secondary data, limitations of the primary trials directly affect the reliability of the findings. Important methodological details—such as oil dosage, selection rationale, and timing of outcome measurement—were often missing, and substratification of the outcome variable varied widely between reviews, preventing any reliable assessment of labour stages, oil choice, or secondary outcomes from being made.

It should be noted as a possible limitation that only publications published after 2000 were considered, even though aromatherapy is a field that has been actively developing for approximately 40–50 years. Most primary studies on aromatherapy were published after 2000. Systematic reviews that predate this might therefore include a very limited number of studies; their inclusion would disproportionately skew the umbrella review results in favour of older research.

Most reviews assessed bias across standard domains (randomization, blinding, attrition, and selective reporting) and found moderate to high risks, particularly regarding blinding, which is inherently challenging in aromatherapy research due to scent detectability. Concerning safety, reviewers generally stress allergic reactions as one possible adverse effect. Though no such reactions were described in the body of the literature at hand, it is possible they have been missed, as not all primary studies treated allergic reactions or controlled for these separately. This umbrella review is thus unable to reach a conclusive safety assessment of aromatherapeutic interventions during labour.

The methodological quality of the included reviews was also limited, with AMSTAR 2 ratings indicating low or critically low confidence in most. Common weaknesses included a risk of publication bias, conflicts of interest from uninvestigated funding sources, incomplete reporting, inconsistent or improperly disclosed inclusion criteria, and limited language and database coverage. Non-English studies were often excluded, reducing comprehensiveness. In addition, language barriers in publication lead to differences in understanding and interpretation. Furthermore, considerable clinical and methodological heterogeneity across trials—especially in essential oil type, application method, and outcome measurement—diminished the strength of the pooled results. Effect sizes calculated by the seven meta-analyses varied widely from small to large. Their comparison was complicated because some authors only used raw mean differences; even if SMDs were provided, it was sometimes unclear which specific statistic was used. Last but not least, the results of primary studies may overlap in the various reviews. This may lead to an overrepresentation of these primary studies’ results and, therefore, introduce further bias into this umbrella review’s literature sample. Nevertheless, the impact of the overlap remains unclear since no formal overlap analysis was conducted, and therefore, consistency across reviews should not be interpreted as robust confirmation. The number of unique primary studies in the reviewed studies ranged from 0% to 50% (Appendix A.2).

Umbrella reviews themselves are constrained by their reliance on previously synthesized data and therefore cannot generate new evidence. They are also vulnerable to bias accumulation, reporting errors, and rapid obsolescence as new reviews emerge. Despite these limitations, this study mitigated bias through repeated literature searches, adherence to PRISMA and PICO guidelines, and the use of AMSTAR 2 for structured quality assessment. However, the involvement of a single reviewer introduces potential subjectivity in study selection and data extraction. Replicating this study with a standard two-reviewer selection and data extraction protocol would help to decrease the risk of erroneously reported data and could strengthen the credibility of the inclusion or exclusion decision-making. This study was limited to PubMed and Google Scholar, which represent only two of many potential databases. It is therefore possible that relevant systematic reviews were not identified, heightening the need for more expansive reviews to be conducted. Google Scholar specifically harbours difficulty for the replication of search strategies; this may hinder efforts to verify or expand on this study’s findings.

In summary, while the included studies collectively suggest aromatherapy may offer benefits in labour pain management, these conclusions must be interpreted cautiously. The evidence base is limited by methodological weaknesses, regional concentration, and inconsistent reporting. Effect size remains unclear, and the current literature in the field is highly heterogeneous. Furthermore, it remains unclear whether the current study distorted the state of research because only reviews that were identifiable in PubMed and Google Scholar were included. Reviews with high or low evidence may therefore have been overlooked. Nonetheless, this umbrella review provides a comprehensive and transparent synthesis of current knowledge, offering valuable direction for future research and practice in evidence-based midwifery care.

## 5. Conclusions

Aromatherapy may reduce labour pain, but the certainty of the evidence is low due to methodological limitations of existing reviews and primary trials. While there is a consensus towards its effectiveness among the literature sample examined in this study, aromatherapy remains generally disputed in the scientific community. This is also due to the fact that the safety of the procedure cannot yet be assessed due to inconsistent and insufficient reporting of possible side effects, which relativizes the suggested positive effects. Based on the available evidence, strong recommendations cannot be made; if used during labour, experience with the practice is required to avoid rare adverse effects. Patch testing the essential oils on a small area of forearm skin before general application is recommended to detect possible allergic reactions. It is highly recommended that guidelines on aromatherapy in midwifery be developed to ensure responsible practice, which should always include sensitivity testing and informed consent from the patient.

## Figures and Tables

**Table 1:** Essential oils commonly used during labour, their effects, and indications.

Name	Effect	Indication
Lavender *Lavandula angustofolia*	Soothing, analgesic, antispasmodic [8]	Pregnancy contractions, labour pain, insomnia, anxiety [8,9,10]
Verbena/ Lemongrass *Verbena officinalis*	Calming, anti-inflammatory, analgesic, uterotonic [8]	Fatigue, anxiety, labour augmentation [8]
Clove *Syzygium aromaticum*	Analgesic, uterotonic [8]	Labour induction [8], labour pain [10]
Rose *Rosa damascena*	Relaxing, antispasmodic, balancing [8]	Anxiety, engagement contractions, afterpains [8], labour pain [10], post-operative pain after C-section [11]
Geranium *Pelargonium graveolens*	Relaxing, uplifting [8]	Anxiety, mood swings, postpartum recovery [8]
Ylang-Ylang *Cananga odorata*	Calming, relaxing [8]	Emotional regulation during labour [8]
Clary sage *Salvia sclarea*	Analgesic, uterotonic, physically and mentally relaxing [12,13,14]	Labour pain [10], labour augmentation [15]
Roman chamomile *Chamaemelum nobile*	Soothing, calming, balancing, increases pain tolerance [12] antispasmodic, analgesic, relaxing [14]	Labour pain [10], often mixed with other oils [15]
Jasmine *Jasminum grandiflorum*	Euphoric, stimulating, antispasmodic, anxiolytic [14]	Labour support, strengthens intuition [12]

**Table 2:** Descriptive data of recruited reviews, part 1.

Reference	Design	Location	Recruited Trials	Population
[24]	Systematic review	Iran, all trials from Iran	33 trials, design unspecified	No restrictions, but n = 30 of the primary studies limited to primiparae
[25]	Systematic review and meta-analysis	Taiwan, most trials from middle east	17 RCTs	Low risk, not limited by parity
[23]	Systematic review and meta-analysis	Taiwan, most trials from the Middle East	8 RCTs, 1 quasi-experimental study	Primigravidae at term with a singleton pregnancy, cephalic presentation, and spontaneous true labour in the first stage of labour
[26]	Systematic review and meta-analysis	Turkey, most trials from middle east	14 RCTs	Low-risk pregnant women between the ages of 18 and 35, at term, cephalic presentation, in active labour
[27]	Systematic review and meta-analysis	Iran, most trials from middle east	21 RCTs, 4 quasi-experimental studies, 1 controlled trial, 1 experimental study	Low-risk women with singleton pregnancies and cephalic presentation who did not use pharmaceutical analgesics or epidurals
[28]	Systematic review and meta-analysis	Turkey, most trials from middle east	10 RCTs	Primiparous pregnant women “who would give birth for the first time” (p. 1261)
[29]	Systematic review and meta-analysis	Iran All trials from Iran	5 RCTs, only 4 of them included in meta-analysis	All women in active labour
[30]	Systematic review and meta-analysis	Japan, most trials from middle east	6 RCTs, 4 quasi-RCTs	Pregnant women at term with labour onset
[31]	Cochrane review (could not perform meta-analysis)	Australia, trials from Italy and New Zealand	2 RCTs	All labouring women

n = number of trials, RCT = randomized controlled trial.

**Table 3:** Descriptive data of recruited reviews, part 2.

Reference	Application Methods	Essential Oils
[24]	Inhalation (n = 23), massage (n = 7), footbath and inhalation (n = 2), unspecified (n = 1)	Lavender, rose, geranium, jasmine, frankincense, peppermint, chamomile, sweet orange, neroli, clove, mandarin. Number of occurrences unspecified.
[25]	Inhalation (n = 9), massage (n = 6), footbath and inhalation (n = 1), unspecified (n = 1)	Lavender (n = 9), jasmine (n = 1), clary sage (n = 1), rose (n = 2), citrus (n = 1), mixed (n = 1), choice (n = 2)
[23]	Inhalation (n = 8), massage (n = 1)	Rose (n = 1), lavender (n = 2), neroli (n = 2), orange (n = 1), geranium (n = 1), jasmine (n = 1), choice (n = 1)
[26]	Inhalation or massage. Number of occurrences unspecified.	Lavender (n = 8), jasmine (n = 3), frankincense (n = 1), rose (n = 1), neroli (n = 1)
[27]	Inhalation (n = 20), massage (n = 7)	Lavender (n = 13), rose (n = 5), mix (n = 6), frankincense (n = 1), olive (n = 2), neroli (n = 1), clary sage (n = 1), peppermint (n = 1)
[28]	Inhalation (n = 6), massage (n = 4), both (n = 1)	Lavender (n = 6), rose (n = 1), jasmine (n = 1), unspecified (n = 1)
[29]	Inhalation (n = 2), massage (n = 2), cold water immersion (n = 1)	Lavender (n = 5)
[30]	Inhalation (n = 10)	Lavender (n = 3), clary sage (n = 1), jasmine (n = 1), rose (n = 2), neroli (n = 2), geranium (n = 1), frankincense (n = 1), choice (n = 1)
[31]	Choice (n = 1) Bath (n = 1)	Choice (n = 1), ginger compared to lemongrass (n = 1)

n = number of trials.

**Table 4:** Descriptive data of recruited reviews, part 3.

Reference	Control	Primary Outcome	Secondary Outcomes
[24]	Standard care, massage w/o EO, footbath w/o EO, biofeedback therapy. Number of occurrences unspecified.	Pain intensity at 30–60 min after intervention via Visual Analogue Scale (VAS)	Anxiety via State–Trait Anxiety Inventory (STAI)
[25]	Standard care (n = 5), inhalation w/o EO (n = 5), massage w/o EO (n = 4), breathing technique (n = 1), unspecified (n = 2)	Pain intensity via ten-point Likert scale or Numerical Rating Scale (NRS); labour duration	Incidence of emergency caesarean section and spontaneous labour events
[23]	Inhalation w/o EO (n = 7), massage w/o EO (n = 1), standard care (n = 1)	Pain intensity via VAS Anxiety via STAI	Apgar scores at 1 and 5 min postpartum
[26]	Inhalation w/o EO, massage w/o EO, standard care. Number of occurrences unspecified.	Labour pain intensity via Numerical Pain Rating Scale (NPRS) (n = 5) or VAS (n = 9)	None
[27]	Standard care (n = 5), inhalation w/o EO (n = 14), breathing technique (n = 1), massage w/o EO (n = 3), unspecified (n = 4)	Labour pain intensity via NRS or VAS	Duration of labour stages Apgar scores at one and five minutes post-natum
[28]	Inhalation w/o EO (n = 4) massage w/o EO (n = 3) Entonox gas (n = 1) Unspecified (n = 2)	Labour pain via VAS	Duration of labour stages Anxiety Apgar scores
[29]	Massage w/o EO (n = 2), inhalation w/o EO (n = 1), cold water immersion w/o EO (n = 1), standard care (n = 1)	Labour pain via VAS	None
[30]	Standard care (n = 2), inhalation w/o EO (n = 7) or breathing technique w/o EO (n = 1)	Labour pain intensity via VAS or NRS Anxiety via STAI or Visual Analogue Scale for Anxiety (VASA)	Duration of contraction, duration of labour, Apgar scores, delivery mode, labour augmentation
[31]	Standard care (n = 1), active control (n = 1)	Various	Various

n = number of trials, w/o = without.

**Table 5:** Main results of included reviews.

Reference	Main Results
[24]	AT was found to be an effective treatment for pain and anxiety during labour in 30 of 33 trials; evidence is most robust for lavender and rose, with studies comparing the two favouring the effect of lavender. Effect sizes not reported.
[25]	AT significantly reduces labour pain during the transition phase (MD: –0.82, 95% CI: –1.55 to –0.09; I^2^ = 93%) and overall (MD: –2.01, 95% CI: –3.63 to –0.39; I^2^ = 96%) No effect on the overall labour duration No effect on maternal and foetal safety measures
[23]	Statistically significant pain reduction in the latent phase (MD: –1.88, 95% CI: –2.98 to –0.78, *p* = 0.0008), early active phase (MD: –1.78, 95% CI: –2.83 to –0.72, *p* = 0.001), and late active phase (MD: –1.72, 95% CI: –2.69 to –0.76, *p* = 0.0004) Significant anxiety reduction in the latent stage (MD: –9.29, 95% CI: –15.88 to –2.69, *p* = 0.006) No statistically significant reduction when compared to base level pain (MD: –0.67, 95% CI: –2.49 to 1.16, *p* = 0.47) and anxiety scores (MD: –5.64, 95% CI: –16.00 to 4.71, *p* = 0.29) Sub-analysis of lavender inhalation only shows a significant effect on latent stage pain level (MD: –1.18, 95% CI: –2.04 to –0.31, *p* = 0.008) with only moderate heterogeneity (I^2^ = 54%, *p* = 0.14)
[26]	AT significantly reduces labour pain with a large total effect size (Hedge’s g: −0.77, *p* < 0.050), though heterogeneity was high (Q = 389.20; *p* = 0.000; significant at the 0.05 level, I^2^ = 96.66%) 11 of 14 RCTs showed a significant analgesic effect
[27]	AT significantly reduces labour pain with a large effect size (SMD: –1.61, 95% CI: –2.08 to –1.14, *p* < 0.00001) Inhalation-based aromatherapy (SMD: –1.73, 95% CI: –2.34 to –1.13) was more effective than massage-based approaches (SMD: –1.24, 95% CI: –1.94 to –0.55)
[28]	AT - significantly reduced labour pain (SMD –0.68, 95% CI: –0.76 to –0.60; Z = 16.32, *p* < 0.01) - significantly shortened labour duration (SMD: –0.36, 95% CI: –0.47 to –0.25; Z = 6.40, *p* < 0.00001) - significantly decreased anxiety levels (SMD: –15.89, 95% CI: –16.78 to –14.99; Z = 34.7) Effects were consistent across labour stages.
[29]	Lavender AT significantly reduced labour pain (SMD −1.05, 95% CI: 0.552–1.548; *p* = 0.000036) with very low heterogeneity (Cochrane Q value = 0.266, *p* = 0.86, and I^2^ = 0%)
[30]	Inhalation AT - reduces labour pain in latent (MD −1.56, 95% CI −2.45 to −0.67, *p* = 0.0006, I^2^ =97%) and early active stage (MD −1.69, 95% CI −2.50 to −0.89, *p* < 0.0001, I^2^ = 96%) - reduces anxiety in early active (SMD −3.49, 95% CI −6.28 to −0.69, *p* = 0.01, I^2^ = 99%) and late active stage (SMD −5.54, 95% CI −10.39 to −0.69, *p* = 0.03, I^2^ = 99%) - shortens the duration of the first stage of labour (SMD −0.21, 95% CI −0.37 to −0.06, *p* = 0.008, I^2^ = 56%), but not of the second stage - did not influence Apgar scores, delivery mode, or the need for labour augmentation
[31]	Insufficient evidence on any effects of aromatherapy during labour

AT = aromatherapy, (S)MD = (standardized) mean difference, CI = confidence interval, I^2^ = heterogeneity measurement, *p* = probability value, (Cochrane), Q = heterogeneity measurement, Z = Z statistic.

**Table 6:** AMSTAR 2 score sheet of all included reviews.

Ref.	1	2 *	3	4 *	5	6	7 *	8	9 *	10	11 *	12	13 *	14	15 *	16	Confidence Level
[24]	N	Y	N	(Y)	Y	Y	N	N	Y	N	-	-	Y	Y	-	Y	Low
[26]	Y	(Y)	N	(Y)	?	N	N	N	Y	N	Y	Y	Y	Y	Y	Y	Low
[28]	Y	Y	N	(Y)	Y	Y	N	(Y)	Y	Y	Y	Y	Y	Y	N	Y	Critically low
[27]	Y	(Y)	N	(Y)	Y	Y	N	Y	Y	N	N	Y	Y	Y	Y	Y	Critically low
[29]	Y	N	N	(Y)	Y	Y	N	Y	Y	N	Y	Y	Y	Y	N	Y	Critically low
[25]	N	Y	N	Y	?	Y	N	(Y)	Y	N	Y	N	Y	Y	N	Y	Critically low
[23]	N	(Y)	N	Y	Y	Y	Y	Y	Y	N	Y	Y	Y	Y	N	Y	Low
[30]	Y	Y	Y	(Y)	Y	N	N	Y	Y	N	Y	Y	Y	Y	N	N	Critically low
[31]	Y	Y	Y	Y	Y	Y	Y	Y	Y	Y	Y	Y	Y	Y	Y	Y	High
Total	6/9	8/9	2/9	9/9	6/9	7/9	2/9	7/9	9/9	2/9	7/8	7/8	9/9	9/9	3/8	8/9	

Y = Yes, (Y) = partial yes, N = No, ? = unclear, - = not applicable. Critical domains are marked by an asterisk (*).

## Data Availability

Data are contained within the article.

## Abbreviations

The following abbreviations are used in this manuscript:

AMSTAR2	A Measurement Tool to Assess Systematic Reviews
APGAR	Variant of Apgar, used as an acronym for appearance, pulse, grimace, activity, and respiration (Newborn Health Assessment Score)
AT	Aromatherapy
CAM	Complementary and alternative medicine
CI	Confidence interval
EO	Essential oil
MD	Mean difference
NRS	Numerical Rating Scale
NRSI	Non-randomized study of interventions
NPRS	Numerical Pain Rating Scale
PICO	Patients, Intervention, Control, Outcome
PRISMA	Preferred Reporting Items for Systematic Reviews and Meta-Analyses
RCT	Randomized controlled trial
SMD	Standardized mean difference
STAI	State–Trait Anxiety Inventory
VAS	Visual Analogue Scale
VASA	Visual Analogue Scale for Anxiety
w/o	without

## Appendix A

### Appendix A.1

**Table A1:** Full List of Excluded Studies and Respective Reasons.

Index	Citation	Reason for Exclusion
PubMed search result 3	Smith CA, Levett KM, Collins CT, Dahlen HG, Ee CC, Suganuma M. Massage, reflexology and other manual methods for pain management in labour. Cochrane Database Syst Rev. 28 March 2018;3(3):CD009290. doi: 10.1002/14651858.CD009290.pub3. PMID: 29589380; PMCID: PMC6494169.	No focus on AT
PubMed search result 5	Hu Y, Lu H, Huang J, Zang Y. Efficacy and safety of non-pharmacological interventions for labour pain management: A systematic review and Bayesian network meta-analysis. J Clin Nurs. December 2021;30(23-24):3398-3414. doi: 10.1111/jocn.15865. Epub 1 June 2021. PMID: 34075656.	No focus on AT
PubMed search result 6	Freeman M, Ayers C, Peterson C, Kansagara D. Aromatherapy and Essential Oils: A Map of the Evidence. Washington (DC): Department of Veterans Affairs (US); September 2019. PMID: 31851445.	No focus on labour pain
PubMed search result 7	Bertone AC, Dekker RL. Aromatherapy in Obstetrics: A Critical Review of the Literature. Clin Obstet Gynecol. 1 September 2021;64(3):572–588. doi: 10.1097/GRF.0000000000000622. PMID: 33927109.	No focus on labour pain
PubMed search result 11	Smith CA, Collins CT, Cyna AM, Crowther CA. Complementary and alternative therapies for pain management in labour. Cochrane Database Syst Rev. October 2006 18;2006(4):CD003521. doi: 10.1002/14651858.CD003521.pub2. PMID: 17054175; PMCID: PMC6984441.	No focus on AT
PubMed search result 12	Smith CA, Collins CT, Cyna AM, Crowther CA. Complementary and alternative therapies for pain management in labour. Cochrane Database Syst Rev. 2003;(2):CD003521. doi: 10.1002/14651858.CD003521. Update in: Cochrane Database Syst Rev. 18 October 2006;(4):CD003521. doi: 10.1002/14651858.CD003521.pub2. PMID: 12804474.	No focus on AT
PubMed search result 13	Di Vito M, Cacaci M, Martini C, Barbanti L, Mondello F, Sanguinetti M, Mattarelli P, Bugli F. Is aromatherapy effective in obstetrics? A systematic review and meta-analysis. Phytother Res. May 2021;35(5):2477–2486. doi: 10.1002/ptr.6975. Epub 9 December 2020. PMID: 33300141.	No focus on labour pain
PubMed search result 14	Lima-De-La-Iglesia C, Magni E, Botello-Hermosa A, Guerra-Martín MD. Benefits of Complementary Therapies During Pregnancy, Childbirth and Postpartum Period: A Systematic Review. Healthcare (Basel). 9 December 2024;12(23):2481. doi: 10.3390/healthcare12232481. PMID: 39685103; PMCID: PMC11640780.	No focus on AT, no focus on labour pain
PubMed search result 15	Nascimento JC, Gonçalves VSS, Souza BRS, Nascimento LC, Carvalho BMR, Ziegelmann PK, Goes TC, Guimarães AG. New approaches to the effectiveness of inhalation aromatherapy in controlling painful conditions: A systematic review with meta-analysis. Complement Ther Clin Pract. November 2022;49:101628. doi: 10.1016/j.ctcp.2022.101628. Epub 28 June 2022. PMID: 35792408.	No focus on labour pain
PubMed search result 16	Berg T, Flunkert S, Brenner E. Systematische Übersichtsarbeit über die individuellen biopsychosozialen Aspekte von Interventionen während einer physiologischen Geburt bei Erstgebärenden [Systematic review of individual biopsychosocial aspects of interventions during a physiological birth in primiparous women]. Z Geburtshilfe Neonatol. April 2025;229(2):131–146. German. doi: 10.1055/a-2506-9511. Epub 12 February 2025. PMID: 39938571.	No focus on AT, no focus on labour pain
PubMed search result 17	Leach S. Aromatherapy massage: an essential service? Pract Midwife. 2006 Mar;9(3):32, 34-5. PMID: 16562658.	Not a systematic review or meta-analysis
Google scholar search result 1	Satya, M. C. N. (2023). Literature Review: Terapi Komplementer untuk Mengurangi Nyeri Persalinan di Berbagai Negara. SEHATMAS: Jurnal Ilmiah Kesehatan Masyarakat, 2(2), 413–424.	No full text available in English or German
Google scholar search result 2	Mahesi, N., Indahwati, L., & Fransiska, R. D. (2023). LITERATURE REVIEW: EFEKTIVITAS PEMBERIAN AROMATERAPI LAVENDER SECARA INHALASI DAN MASASE TERHADAP PENURUNAN NYERI PERSALINAN KALA I. Journal of Nursing Care and Biomolecular, 8(1), 84–97.	No full text available in English or German
Google scholar search result 3	Ernawati, S., Maolinda, W., & Anisa, F. N. (2021, July). Pengaruh Aromaterapi Lavender Terhadap Nyeri Persalinan: Literatur Review: The Effect Of Lavender Aromatherapy On Labor Pain: Literature Review. In Proceedings of Sari Mulia University Midwifery National Seminars (Vol. 3, No. 1, pp. 217–225).	No full text available in English or German
Google scholar search result 4	Maddocks, W. (2023). Aromatherapy in nursing and midwifery practice: A scoping review of published studies since 2005. Journal of Holistic Nursing, 41(1), 62–89.	No focus on labour pain, not a systematic review or meta-analysis
Google scholar search result 5	Bertone, A. C., & Dekker, R. L. (2021). Aromatherapy in obstetrics: a critical review of the literature. Clinical Obstetrics and Gynecology, 64(3), 572–588.	No focus on labour pain, not a systematic review or meta-analysis
Google scholar search result 6	Yakoeb, A. R., Fitriana, F., Yulivantina, E. V., & Ernawati, E. The Effectiveness of Giving Lavender (Lavandula Angustifolia) Aromatherapy to Reduce Labor Pain: A Systematic Literature Review. Journal of Health, 9(1), 17–23.	No full text available in English or German
Google scholar search result 7	Van Rooyen, R. M. (2021). The use of essential oils for pain relief and anxiety during childbirth: a systematic review (Doctoral dissertation, North-West University (South-Africa)).	Grey literature
Google scholar search result 8	Ghiasi, A., Bagheri, L., & Sharaflari, F. (2022). Effectiveness of aromatherapy in reducing duration of labour: a systematic review. Journal of Obstetrics and Gynaecology, 42(7), 2573–2582.	No focus on labour pain
Google scholar search result 10	Shaterian, N., Pakzad, R., Fekri, S. D., Abdi, F., Shaterian, N., & Shojaee, M. (2022). Labor pain in different dilatations of the cervix and Apgar scores affected by aromatherapy: a systematic review and meta-analysis. Reproductive Sciences, 1–17.	Duplicate

### Appendix A.2

**Table A2:** Proportion of Unique Primary and Overlapping Trials in the Studies.

Reference	Number of Trials	Proportion of Unique Primary Trials: n (%)	Proportion of Overlapping Trials: n (%)
[23]	9	2 (22%)	7 (78%)
[24]	33	14 (42%)	19 (58%)
[25]	17	2 (12%)	15 (88%)
[26]	14	2 (14%)	12 (86%)
[27]	27	8 (30%)	19 (70%)
[28]	10	3 (30%)	7 (70%)
[29]	5	1 (20%)	4 (80%)
[30]	10	0 (0%)	10 (100%)
[31]	2	1 (50%)	1 (50%)
